# Phenotypic and genetic characterization of *Dunaliella* (Chlorophyta) from Indian salinas and their diversity

**DOI:** 10.1186/2046-9063-8-27

**Published:** 2012-11-01

**Authors:** Krishna Preetha, Lijo John, Cherampillil Sukumaran Subin, Koyadan Kizhakkedath Vijayan

**Affiliations:** 1 Genetics and Genomics Section, Marine Biotechnology Division, Central Marine Fisheries Research Institute, Post Box No. 1603, Ernakulam North P.O, Kochi, 682018, India

**Keywords:** Dunaliella, Diversity, India, 18S rDNA, ITS, *rbc*L gene

## Abstract

**Background:**

The genus *Dunaliella* (Class – Chlorophyceae) is widely studied for its tolerance to extreme habitat conditions, physiological aspects and many biotechnological applications, such as a source of carotenoids and many other bioactive compounds*.* Biochemical and molecular characterization is very much essential to fully explore the properties and possibilities of the new isolates of *Dunaliella*. In India, hyper saline lakes and salt pans were reported to bloom with *Dunaliella* spp. However, except for the economically important *D. salina,* other species are rarely characterized taxonomically from India. Present study was conducted to describe *Dunaliella* strains from Indian salinas using a combined morphological, physiological and molecular approach with an aim to have a better understanding on the taxonomy and diversity of this genus from India.

**Results:**

Comparative phenotypic and genetic studies revealed high level of diversity within the Indian *Dunaliella* isolates. Species level identification using morphological characteristics clearly delineated two strains of *D. salina* with considerable *β-*carotene content (>20 pg/cell)*.* The variation in 18S rRNA gene size, amplified with MA1-MA2 primers, ranged between ~1800 and ~2650 base pairs, and together with the phylogeny based on ITS gene sequence provided a pattern, forming five different groups within Indian *Dunaliella* isolates. Superficial congruency was observed between ITS and *rbc*L gene phylogenetic trees with consistent formation of major clades separating Indian isolates into two distinct clusters, one with *D. salina* and allied strains, and another one with *D. viridis* and allied strains. Further in both the trees, few isolates showed high level of genetic divergence than reported previously for *Dunaliella* spp. This indicates the scope of more numbers of clearly defined/unidentified species/sub-species within Indian *Dunaliella* isolates.

**Conclusion:**

Present work illustrates Indian *Dunaliella* strains phenotypically and genetically, and confirms the presence of not less than five different species (or sub-species) in Indian saline waters, including *D. salina* and *D. viridis*. The study emphasizes the need for a combined morphological, physiological and molecular approach in the taxonomic studies of *Dunaliella*.

## Background

*Dunaliella,* the unicellular microalga, is one of the best studied organisms in both general and applied phycology for its higher tolerance to extreme conditions of salinity, light, temperature and pH, as well as for its richness in natural carotenoids, glycerol, lipids and many other bioactive compounds [1-4]. *Dunaliella salina* is reported as the most halotolerantt photosynthetic eukaryote with a remarkable degree of tolerance from 0.5 to 5 M salt concentrations (30–300 ppt) [2]. This genus naturally inhabits saline and hypersaline waters and has a cosmopolitan distribution [5] and of the 28 species of *Dunaliella*, 23 are saline or hypersaline [3,6-9].

Many countries, including India, use *D. salina* for the industrial production of *β-*carotene with wide range of applications [4,10-12]. Apart from *D. salina*, *D. tertiolecta* is used in aquaculture, while many other species were found promising for the production of biofuel, bioprospecting of antioxidants, bioactive compounds etc. [4,6]. Considering the economic importance, most of the studies were mainly focused on the taxonomic, physiological and biotechnological aspects of the halophilic species *D. salina*[5,13-19] (especially from Indian subcontinent) and on the marine species *D. tertiolecta*. But similar exclusive or comparative studies are rarely available for other species [20-23], probably due to their lesser importance and/or limited distribution.

Typically the taxonomy of *Dunaliella* anchors on the morphological and physiological features of the organism. Apart from the general morphology, salinity tolerance and carotenoid (especially *β*-carotene) production are the two commonly studied physiological attributes of *Dunaliella*, where considerable variations have been accounted at inter and intra-species levels [24,25]. Recently, Borowitzka and Siva [3] have given a detailed account of taxonomic revision of the genus *Dunaliella* with special emphasis on saline species bringing more clarity in classification. *Dunaliella* are unique in having a thin plasma membrane instead of a rigid cell wall [26] and are able to change their cell shape and volume in response to changes in osmolarity and other growth conditions [17,27-29]. Due to this high plasticity of cell morphology, the traditional practice of species differentiation merely based on light microscopic observations becomes difficult and time consuming. Consequently many misidentifications arose in the literature which brought in controversies and confusions in the taxonomic organization of the genus *Dunaliella*[3,5].

Molecular taxonomy emerged as a faster and powerful tool as it is consistent and independent from environmental factors and growth stages [30]. It seems to be an advanced and reliable device for the characterization and differentiation of morphologically plastic organisms. Since 1999, molecular characterization has been found promising in the taxonomy of *Dunaliella*[6,29]. Currently 18S rRNA gene [5,31], Internal Transcribed Spacer (ITS) region [14,23,27] and large subunit of the ribulose-bisphosphate carboxylase (*rbc*L) gene [9] are being widely used as effective molecular tools in *Dunaliella* characterization and biodiversity studies. Use of these molecular markers has resulted in the re-designation of many species [8,32]. Nevertheless, the confusion regarding the taxonomy still persists due to the misidentifications, and will be there until a complete revision is made, using an integrated approach with molecular phylogeny, supported by morphological and physiological attributes. Many researchers opined to have such an integrated approach rather than a single system of taxonomic identification [3,32].

In India, *Dunaliella* are found in salt pans, saline and hypersaline ponds, lakes, pools etc. as a major primary producer. Many species including *D. salina* have been reported [33] to form blooms in salt pans. However, taxonomic characterization of Indian *Dunaliella* strains based on phenotypic and molecular traits is rarely available. In this background, we conducted a study on the characterization of *Dunaliella* strains isolated from the Indian salinas, using morphological, physiological and molecular tools and have made an attempt to depict the best possible description on Indian *Dunaliella* spp. Based on the results obtained, taxonomic position and diversity aspects of the Genus *Dunaliella* from Indian salinas are discussed.

## Results and discussion

### Morphological & physiological parameters

**Figure 1:**
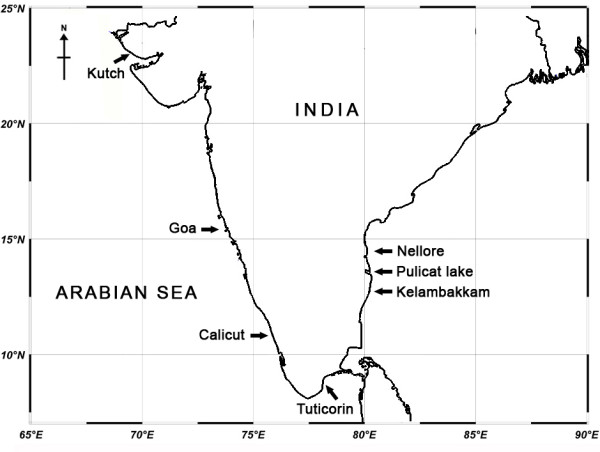
**Sampling locations of *Dunaliella* strains used in the present study along the Indian coast.**

**Figure 2:**
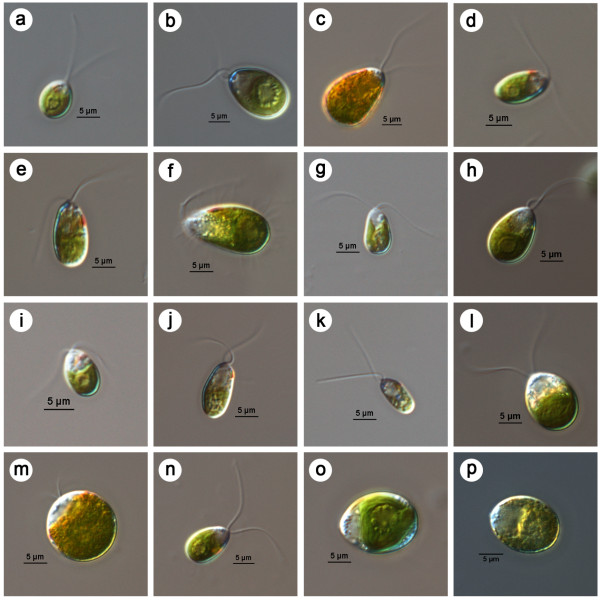
**DIC microscopic images of different *Dunaliella* isolates.** (**a**) *Dunaliella sp*. S086 (Tuticorin salt pan), (**b**) & (**c**) *D. salina* S089 (CMFRI old strain), (**d**) *D. viridis*? S115 (Chennai salt pan), (**e**) & (**f**) *Dunaliella* sp. S118 (Nellore salt pan), (**g**) & (**h**) *Dunaliella sp.* S121 (Pulicat lake), (**i**) *D. viridis*? S122 (Goa salt pan), (**j**) *Dunaliella* sp. S125 (Goa salt pan), (**k**) *Dunaliella* sp. S133 (Kutch salt pan), (**l**) & (**m**) *D. salina* S135 (Calicut marine isolate), (**n**) *Dunaliella* sp. S147 (Kutch salt pan), (**o**) & (**p**) *D. salina* CS265 (Australian reference strain). In brackets given the origin of isolates. (**c**) & (**m**) orange red cells of Indian isolates of *D. salina* (S089 & S135) grown at 4.5 M NaCl concentration. (**f**) & (**h**) large yellow green cells of S118 and S121 at 4.5 M NaCl. (**p**) Reference strain *D. salina* CS265 at 2.5 M NaCl turning orange. Scale bar given – 5 μm.

Morphologically all ten strains of the green biflagellate chlorophytes, isolated from 7 different locations along the Indian coast (Figure 1 & Table 1), were identified as *Dunaliella* (Figure 2) following the revision of the genus by Borowitzka and Siva [3]. Of the 10 strains, 9 were isolated from hypersaline water bodies and 1 (S135) was marine. Though purified by agar plating, the cultures were not axenic. All morphological characteristics of different geographical Indian isolates of *Dunaliella* are summarized in Table 2.

**Table 1:** **Geographical origin and gene sequence accession details of*Dunaliella*strains studied in the present work**

**Groups**	**Strain code**	**Isolated from**	**Geographic co-ordinates**	**Month of collection**	**Salinity of the sampled water**	**18S rDNA product size**	**Genebank accession No.**		
							**18S rDNA**	**ITS region**	***rbc*L gene**
	CS265	*Dunaliella salina*; Reference strain from CSIRO collection of living microalgae, Australia				2210 bp	JN807321	JN797804	JN797820
I	MBTD-CMFRI-S135	Sea water, Calicut, Kerala (WC)	11°15’ N 75°46’ E	May 2009	33 ppt	2230 bp	JF708161	JN797802	JN797818
	MBTD-CMFRI-S089	Kelambakkom salt pan, Chennai, TN (EC)	Culture maintained in CMFRI phytoplankton culture collection, isolated from Chennai salt pan.			2210 bp	JF708173	JN797806	JN797811
II	MBTD-CMFRI-S118	Salt pan, Nellore, AP (EC)	14°16’ N 80°07’ E	March 2009	300 ppt	2290 bp	JN807316	JN797808	JN797813
	MBTD-CMFRI-S086	Salt pan, Tuticorin, TN, (EC)	08°47’ N 78°09’ E	February 2009	300 ppt	2290 bp	JF708169	JN797805	JN797810
	MBTD-CMFRI-S121	Pulicat salt lake, AP (EC)	13°40’ N 80°11’ E	March 2009	150 ppt	2250 bp	JN807317	JN797809	JN797814
III	MBTD-CMFRI-S115	Kelambakkom salt pan, Chennai, TN (EC)	12°45’ N 80°12’ E	March 2009	380 ppt	2550 bp	JN807315	JN797807	JN797812
	MBTD-CMFRI-S122	Salt pan, Ribandar, Goa (WC)	15°30’ N 73°51’ E	May 2009	280 ppt	2550 bp	JN807318	JN797799	JN797815
	MBTD-CMFRI-S133	Salt pan, Kutch, Gujarat (WC)	23°50’ N 69°39’ E	July 2009	320 ppt	2530 bp	JF708183	JN797801	JN797817
IV	MBTD-CMFRI-S125	Salt pan, Pilar, Goa (WC)	15°26’ N 73°53’ E	May 2009	260 ppt	2640 bp	JN807319	JN797800	JN797816
V	MBTD-CMFRI-S147	Salt pan, Kutch, Gujarat (WC)	23°50’ N 69°39’ E	April 2009	180 ppt	1820 bp	JN807320	JN797803	JN797819

NB: For convenience strain codes used in text included only third part of full strain code (e.g., S086). *AP*, Andhra Pradesh; *TN*, Tamil Nadu; WC-west coast; *EC*, east coast.

Indian strains were grouped into subsets based on the 18S rDNA size obtained by PCR amplification with MA1-MA2 primers.

**Table 2:** **Morphological and physiological characteristics of 10 Indian*Dunaliella*strains**

**Groups**	**Strain Code**	**Cell colour**	**Cell shape**	**Flagella length**	**Stigma**	**Pyrenoid**	**Refractile granules**	**Mode of reproduction observed**	***β *Carotene normal/stress (pg/cell)**	**Salinity optimum**	**Identified as**
I	MBTD-CMFRI-S135	Green to red	Ovoid, spherical, cylindrical	1.3 or 1.5 to cell length	Not clearly visible or diffuse	Large with distinct amylosphere	Absent	Cell division	8.68/22.94	1.5M NaCl	*D. salina*
	MBTD-CMFRI-S089	Green to red	Ovoid, spherical	1.3 to cell length	Not visible/ Diffuse large	Large with distinct amylosphere	Absent	Cell division	6.53/23.36	1.5M NaCl	*D. salina*
II	MBTD-CMFRI-S118	Green to orange	Ovoid spherical	1.5 to cell length	One; large, red, median, diffuse	Small with amylosphere	Absent	Sexual, cell division	2.11/3.47	2.5M NaCl	*Dunaliella sp.*
	MBTD-CMFRI-S121	Green	Ovoid pyriform	1.5-2 to cell length	One; large, red, median, distinct	Large with amylosphere	Absent	Sexual, cell division	1.59/2.17	1.5M NaCl	*Dunaliella sp.*
	MBTD-CMFRI-S086	Green	Ovoid, oval or pyriform	1.5- 2 to cell length	One; Small, red, median, distinct	Small with amylosphere	Present	Sexual, cell division	2.68/3.41	0.5 M NaCl	*Dunaliella sp.*
III	MBTD-CMFRI-S115	Green	Ovoid, oval or fusiform	1.3 to cell length	One; small, red, anterior, distinct	Small with amylosphere	Absent	Palmella, aplanospores	1.05/1.99	1.5M NaCl	*D. viridis?*
	MBTD-CMFRI-S122	Green	Oval, cylindrical,	1.3 to cell length	One; large, red, anterior, distinct,	Large, Amylosphere	Present	Palmella stage	0.67/1.78	1.5M NaCl	*D. viridis?*
	MBTD-CMFRI-S133	Yellow green	Fusiform, Elliptical	1.3 to cell length	One, Two at lower salinity; small, red, median, distinct	Small, with amylosphere	Absent	Cell division, Palmella, aplanospores	0.51/1.26	1.5M NaCl	*D.viridis/D. bioculata?*
IV	MBTD-CMFRI-S125	Green	Cylindrical fusiform	Equal or 1.3 to cell length	One, large, red, anterior, distinct	Small, with distinct separate starch grains	Absent	Cell division	0.70/1.8	1.5M NaCl	*D. minuta?*
V	MBTD-CMFRI-S147	Green	Oval, fusiform	1.5 or 2 to cell length	One, large, red, median distinct	Large with amylosphere	Present	Palmella (dominant stage), Cell division	0.89/6.7	1.5M NaCl	*Dunaliella sp.*

Grouping of the subsets was formed based on common morphological features including cell size and *β*-carotene accumulation at high salinity and light (stress).

High levels of morphological plasticity in cell shape and size was observed among all the 10 *Dunaliella*, strains, but a general consistency in cell size was noticed within the range given (Table 3) [34,35]. Among the 10 strains, S135 (Calicut, marine isolate), S089 (CMFRI old strain isolated from Chennai) and S147 (Kutch) were considerably larger while strain S133 (Kutch) was the smallest.

**Table 3:** **Descriptive statistics of cell size variables and F-values (derived from the analysis of variance) of different*Dunaliella*isolates from Indian coast**

**Groups**	**I**		**II**			**III**			**IV**	**V**	
**Strain code**	MBTD-CMFRI-S135	MBTD-CMFRI-S089	MBTD-CMFRI-S086	MBTD-CMFRI-S118	MBTD-CMFRI-S121	MBTD-CMFRI-S115	MBTD-CMFRI-S122	MBTD-CMFRI-S133	MBTD-CMFRI-S125	MBTD-CMFRI-S147	**F value**
**Length** μm	17.51±1.78 (12.30-21.17)	14.12±2.25 (10.01-18.82)	9.15±1.02 (6.44-10.68)	9.51±1.09 (7.96-12.25)	9.37±1.30 (6.45-11.77)	9.02±0.96 (6.79-12.12)	8.46±1.12 (5.62-10.55)	7.91±0.93 (6.54-9.78)	9.89±1.37 (8.38-12.99)	11.17±1.50 (8.02-13.83)	138.33*
**Width** μm	10.30±1.96 (8.61-19.79)	9.57± 1.35 (7.46-12.58)	6.14±0.92 (3.52-8.08)	6.91±0.74 (5.84-8.76)	5.94±0.96 (4.14-7.54)	5.09±0.77 (3.02-6.98)	4.74±0.48 (3.91-5.76)	3.89±0.60 (3.11-5.10)	4.34±0.69 (3.27-5.95)	7.23±1.15 (5.40-10.07)	125.85*

Measurements are presented as, Mean ± SD (min. - max.); *Significant at the 1% level; *SD* is standard deviation.

Grouping of subsets was statistically formed based on the average length/width of the *Dunaliella* cells.

In salinity tolerance study (0.5 – 4.5 M NaCl), sufficient growth (approximately 5–20 million cells/ml in 28 days from an initial cell density of 15–60 thousand cells/ml) was obtained for each strain in different salinities with optimum growth at 1.5 or 2.5 M salt concentrations (growth rate was 0.1±0.05 div.d^-1^ during exponential growth period), emphasizing that all the strains (including the marine isolate S135) are halophilic in nature. Beta carotene was quantified in all the isolates (Table 2) at ‘normal’ and stressed growth conditions. Under stress (3.5M NaCl, irradiance of 100–150 μmol photons m^-2^ s^-1^) higher level of the pigment (23.4 & 22.9 pg/cell) was recorded in the 2 Indian strains, S089 and S135 respectively while for the Australian reference strain *D. salina* CS265, it was nearly 36 pg/cell. The 3 strains turned orange/red at high salinity (Figure 2, c, m & p). Lower quantities of the pigment (<2 pg/cell) were observed in the strain S133 from Kutch and the 2 Goa strains, S122 and S125. For the remaining strains it was around 2–7 pg/cell, under stress.

Among the many listed attributes, cell size, colour, stigma and *β-*carotene accumulation are the major traits used to discriminate carotenogenic *Dunaliella* spp. like *D. salina* and *D. bardawil/salina*. Red *D. salina* (especially at high salinity) was reported to have significantly large cell size than other common strains like *D. parva*, *D. viridis* and *D*. *tertiolecta*[3]. Limited carotenogenic capacity also discriminated other strains from *D. salina* where the latter can accumulate >20 pg *β-*carotene/cell [3]. Coesel et al. [36] and Olmos et al. [5] obtained 10 pg/cell of *β-*carotene under non-stressful growth conditions for the two hyper producing strains of *D. salina*, CCAP 19/30 and 19/18 respectively. In the present study, morphological and physiological observations of the 2 strains, S089 and S135, revealed that they are Indian strains of *D. salina*. Discrimination derived from basic morphology (taxonomic key), characterized the remaining strains as *D. viridis* except S125 (*D. minuta*?), S133 (*D. viridis/D. bioculata?*) and S147 (*Dunaliella sp.)* (Table 2). Detailed morphology and physiology based studies illustrated considerable diversity within the Indian strains of *Dunaliella* but a little confusion prevailed due to overlapping features with more than one reported species (like the cell size increase and *β*-carotene content of strains S121 and S118 at higher salinity, two stigmata of S133 at lower salinity and pyrenoid characteristics of S125) (Tables 2 and 3). In the present investigation, molecular characterization was used as a tool to resolve the confusion.

### Molecular characterization based on 18S rRNA gene size

**Figure 3:**
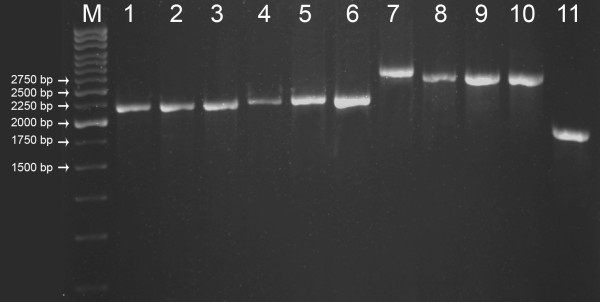
**18S rDNA amplification with MA1 & MA2 primers in 1% Agarose gel.** Lane 1–11 CS265, MBTD-CMFRI-S089, S135, S086, S118, S121, S125, S115, S122, S133 and S147 respectively & Lane M -250bp ladder (Genie, India).

Amplification of 18S rRNA gene with primers MA1 & MA2 from different *Dunaliella* isolates in the present study gave products with size ranging from ~1820–2640 bp (Table 1 & Figure 3). The banding pattern observed among the present isolates was found matching with the reported gene sizes of 18S rDNA using MA1-MA2 primers [5,29]. The dissimilarity in product size observed among the different isolates could be explained based on the presence/absence or difference in the size of introns across different species of *Dunaliella*[5,29]. With regard to the presence of 3 types of Group I introns in 18S rRNA gene [37], Olmos et al., [31,38] designed a set of conserved primers (MA1, MA2 & MA3) and a set of species specific primers (DSs, DPs, DBs). They used the conserved primers (MA1 & MA2) for preliminary differentiation of various known species of *Dunaliella* based on the size of the PCR product. Subsequently, morphologically identical *Dunaliella* strains (e.g., *D. salina* and *D. bardawil*) got discriminated by position and number of the introns [5,29]. Based on these reports 18S rDNA of *D. tertiolecta* (~1770 bp) lacks an intron, *D. salina* (~2170 bp) has only one intron at 5’ terminus, *D. viridis* (~2495 bp) has one longer intron again at 5’ terminus and *D. parva* and *D. bardawil* have two introns (~2570 bp) one each at 5’ and 3’ terminus. Other than these strains, *D. peircei* having ~2088 bp (one intron at 5’ terminus) was also reported.

In the present study, based on the 18S rDNA gene size, clear grouping of all the 10 Indian *Dunaliella* strains was possible (Figure 3 & Table 1). Out of the 10 strains only 1 strain, S147 (Kutch) produced the shortest band (~1820 bp) showing similarity to that reported for *D. tertiolecta* (~1770 bp) probably due to the absence of any introns (Group V). The 2 Indian strains S089 (CMFRI old strain, Chennai) and S135 (Calicut marine isolate) and the reference strain CS265 (*D. salina*) produced ~2200 bp size band (Table 1) closer to the reported *D. salina* (~2170 bp). This further supported phenotypic identification of the above 2 Indian strains as *D. salina* (Group I). Studies using the 18S PCR products revealed a clear separation of morphologically similar strains (*D. viridis?*), into 2 groups - (Group II & Group III in Table 1 & Figure 3). The 18S rDNA size (~2300 bp) of Group II strains (S086 (Tuticorin), S118 (Nellore) & S121 (Pulicat)) was showing an indication that these strains are more close to *D. salina* than *D. viridis*. While group III strains (S122 (Goa), S115 (Chennai) & S133 (Kutch)) gave a band size of ~2550/2530 bp which could be compared to the reported *D. viridis* (2495 bp) or *D. parva* (~2570 bp) probably with 1 or 2 introns. The band size of the Goa strain S125 (~2640 bp, Group IV) was however not in accordance with any of the reported species of *Dunaliella*[5]. Partial (~600 bp) sequencing of 5’ terminus region of the PCR products could not confirm the presence of any introns, while the generated partial sequence information (refer Table 1 for GenBank accessions) was found to be highly conserved across species and therefore could not specify the species delineation. Further characterization was carried out based on molecular phylogeny of a more variable ITS region and a conserved *rbc*L gene for more clarification about species lineages of Indian *Dunaliella.*

### ITS phylogeny

**Figure 4:**
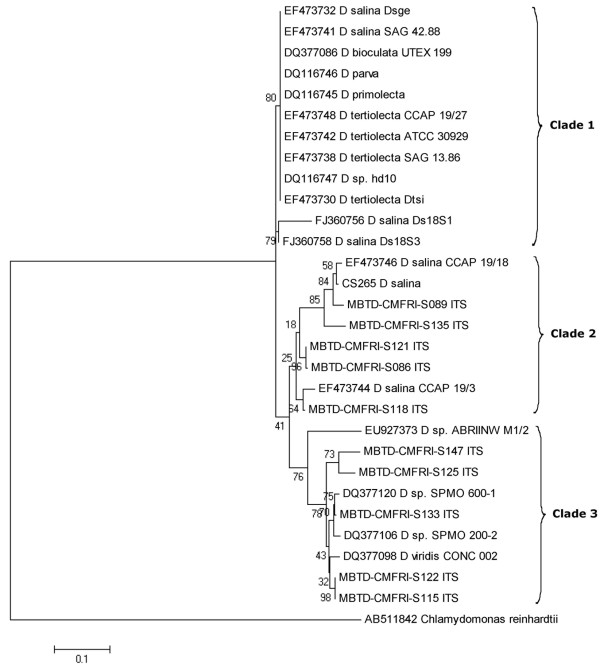
**Phylogenetic tree of the Maximum Likelihood (ML) analysis inferred from the nuclear encoded ITS regions including 5.8S rDNA of *Dunaliella.*** Bootstrap values for 1000 replicates are given at the internal nodes.

**Figure 5:**
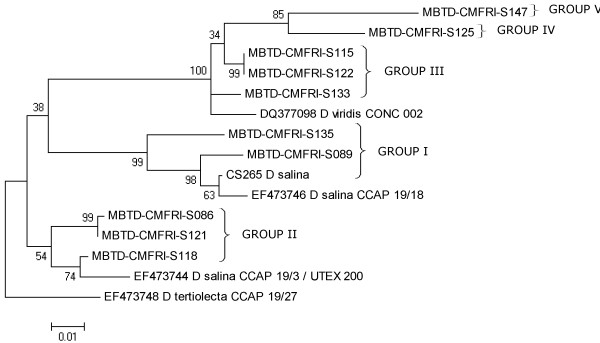
**Phylogenetic tree (ML) of ITS region out grouped with *D. tertiolecta*; illustrated groups of 10 Indian *Dunaliella* strains.** Bootstrap values for 1000 replicates are given at the internal nodes.

The phylogenetic analysis based on ITS region (~700 bp) using maximum likelihood confirmed high level of genetic diversity within Indian *Dunaliella* isolates. All *Dunaliella* spp. (including the sequences from NCBI, Table 4) were found to be separated into 3 major clusters, with *Chlamydomonas reinhardtii* forming an out group as expected (Figure 4). Majority of the named species of *Dunaliella* from NCBI were found to be grouped under a single cluster (clade 1) except *D. salina* CCAP 19/18, *D. salina/D. viridis* CCAP 19/3, *D. viridis* CONC 002 and *Dunaliella* spp. ABRIINW M1/2, SPMO 200–2, SPMO 600–1 and the reference strain of *D. salina* CS265. When out grouped with *D. tertiolecta* (Figure 5), the ITS tree branching was found well supporting the morphology and 18S rRNA gene size based grouping (Group I-V) of the 10 new isolates of *Dunaliella*.

**Table 4:** **Sequence accession no.s of *Dunaliella* and other strains from NCBI database included in present study.**

**Strain**	**ITS Accession No.**	**rbcL Accession No.**
*D tertiolecta* CCAP 19/27	EF473748	
*D tertiolecta* ATCC 30929	EF473742	
*D tertiolecta* SAG 13.86	EF473738	
*D tertiolecta* Dtsi	EF473730	
*D tertiolecta* CCMP 1302		DQ313205
*D tertiolecta* UTEX 999		DQ313203
*D peircei/D viridis* UTEX 2192		DQ313196
*D primolecta*	DQ116745	DQ173090
*D primolecta*		AB127992
*D primolecta* UTEX 1000		DQ313198
*D parva*	DQ116746	
*D parva/D viridis* UTEX 1983		AJ001877
*D bioculata* UTEX 199	DQ377086	DQ313195
*D bioculata*		AB127991
*D salina* Dsge	EF473732	
*D salina* SAG 42.88	EF473741	
*Dunaliella* sp. hd10	DQ116747	
*D salina* Ds18S3	FJ360758	
*D salina* Ds18S1	FJ360756	
*D salina*/*D viridis* CCAP 19/3 (UTEX 200)	EF473744	DQ313197
*D salina* CCAP 19/18	EF473746	GQ250046
*D viridis* CONC 002	DQ377098	DQ313206
*Dunaliella* sp. ABRIINW M1/2	EU927373	
*Dunaliella* sp. SPMO 200-2	DQ377106	DQ313211
*Dunaliella* sp. SPMO 600-1	DQ377120	DQ313218
*Chlamydomonas reinhardtii*	AB511842	
*Paulschulzia pseudovolvox*		D86837

The genetic divergence values observed among clade 2 (Figure 4) isolates ranged up to 9.1% (between S089 & CCAP 19/3), which was comparable to that observed between different species of the genus *Dunaliella*[14,17]. The 2 Indian *D. salina* strains (Group I, Figure 5) S089 (CMFRI old strain) and S135 (Calicut marine isolate) got clustered with the Australian *D. salina* strains CS265 and CCAP 19/18 with divergence values ranging from 1.9% (between CS 265 & CCAP 19/18) to 5.6% (between S089 & S135). Whereas, the strains S086 (Tuticorin), S121 (Pulicat) and S118 (Nellore) were found closer to *D. salina/D. viridis* CCAP 19/3. The much higher divergence (>8%) of the 3 Indian strains from the carotenogenic *D. salina* strains (CCAP19/18 and CS265) was in agreement with the grouping of the 3 strains in Group II (Figure 5) based on the morphological, physiological and 18S rDNA size based analyses.

The remaining 5 Indian *Dunaliella* strains (S115, S122, S125, S133 and S147) along with *D. viridis* CONC 002 formed a separate cluster (clade 3 of Figure 4). The strains showed divergence range from 0% (between S115 & S122) to 7.6% (between S147 and *D. viridis* CONC 002). The 2 *D. viridis(?)* strains S115 (Chennai) & S122 (Goa) and the *D. viridis/D. biocuata(?)* strain S133 (Kutch) were found in close proximity (mean divergence of 2.22%) with CONC 002 *D. viridis* (Group III of Figure 5). The other 2 strains S125 and S147 (Group IV & V of Figure 5) were found to be well separated from the above group with divergence values of 4.98% and 6.42% respectively with *D. viridis* CONC 002.

The mean pair wise genetic distance values observed among the Indian isolates of the 2 major clades (5.35% for clade 2 and 5.12% for clade 3) were comparatively higher than that observed among the named species of *Dunaliella* (1.14% clade 1). Further, the genetic divergence values observed among the Indian *Dunaliella* isolates based on ITS sequence variations were considerably higher than that reported in *Chlamydomonas* spp. (a minimum of 3.5% between 2 species) by Coleman & Mai (1997) [39]. Thus, the pattern of genetic divergence, along with the phylogenetic divergence pattern, clearly indicates the presence of at least 5 or more number of species/sub-species among the 10 Indian strains (including *D. salina* and *D. viridis*).

### *rbc*L gene phylogeny

**Figure 6:**
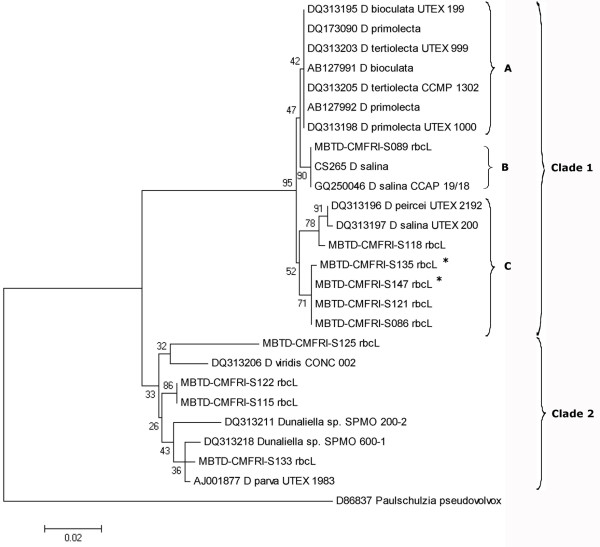
**Phylogenetic tree of the Maximum Likelihood (ML) analysis inferred from the *rbc*L plastid gene partial sequences.** Bootstrap values for 1000 replicates are given at the internal nodes.* show the position changes of S147 and S135 strains.

The pattern of genetic diversity observed among the Indian *Dunaliella* strains based on *rbc*L gene sequence variations was in accordance with the above observations based on 18S rDNA and ITS analysis except for the positioning of S147 and S135. The phylogenetic tree constructed, using maximum likelihood (Figure 6) analysis with *rbc*L gene sequence data, was forming 2 major clusters with *Paulschulzia pseudovolvox* as out group. The mean genetic divergence value observed between the 2 clades was 5.89% and that observed among different isolates of *Dunaliella* ranged from 0.16% to 7.73%.

Being a protein coding gene, the pairwise genetic divergence (Tamura 3 parameter) values observed among *Dunaliella* isolates based on *rbc*L gene sequences were found to be less in comparison with that observed in ITS (a non coding region) sequences. The independent phylogenetic analyses using ITS (Figure 4) and rbcL gene (Figure 6) sequences (Kimura 2 and Tamura 3 parameters respectively) were found to be taxonomically incongruent especially in clade 1. The major topological change observed was the change in the positioning of the isolate S147. Within *rbc*L phylogeny, this strain from Kutch was found closely allied with clade 1 (Figure 6), *w*hereas, with ITS data it was close to *D. viridis* CONC 002 and other Indian isolates (S125, S133, S115 and S122) in clade 3 (Figure 4). Similarly, the marine *D. salina* strain S135, got clustered with non-carotenogenic strains in sub-clade C (with divergence of 1.15%) instead of carotenogenic *D. salina* (sub-clade B).

Clustering of all the remaining 8 strains in both ITS and *rbc*L phylogenies was more or less similar. As expected S089 (*D. salina*, CMFRI old strain) clustered with carotenogenic *D. salina* species CS265 and CCAP 19/18 in clade 1, (sub-clade B, with 100% similarity). The positioning of 3 strains S086, S118 & S121 (sub-clade C, in Figure 6) along with *D. salina/D. viridis* UTEX 200/ CCAP 19/3 and *D. peircei/D. viridis* UTEX 2192 (with <1% divergence value) strongly suggests further taxonomic revision. Similarly, in clade 2, the positioning of the Goa isolate S125 (with maximum divergence 7.33%) and the clustering of strains S133, S122 & S115 with *D. viridis* CONC 002 and *D. parva/D. viridis* UTEX 1983 (with divergence values 3.15% & 0.33% respectively) was in concordance with ITS phylogeny.

### Grouping of Indian *Dunaliella* strains

The 2 larger carotenogenic strains (>20 pg/cell *β-*carotene content) S135 and S089 forming the GROUP I, produced 18S rDNA size ~2200 bp and clustered with *D. salina* CCAP 19/18 and CS265 in ITS phylogeny. These results confirmed the taxonomical identity of the 2 strains as *D. salina* (Section Dunaliella)*.* But closeness of S135 to the 2 morphologically dissimilar, lower *β-*carotene content strains, S121 (Pulicat) and S086 (Tuticorin) in *rbc*L phylogeny has to be noted, which may be due to its marine origin.

GROUP II included the strains S086 (Tuticorin), S118 (Nellore) and S121 (Pulicat), which clustered with *D. salina/D.viridis* (CCAP 19/3) in ITS phylogeny and had ~2300 bp band for 18S rDNA. The present study shows the closeness of these 3 strains to *D. salina* by molecular analysis (18S rDNA size and ITS &*rbc*L phylogenies) rather than by morphological features. These strains were with lesser *β-*carotene content (~2–4 pg/cell) and cells were always green (only S118 turned slightly orange at higher salinity), smaller and with a clear stigma, which were not corresponding with hyper *β-*carotene producer strain of *D. salina* and are more or less characters of *D. viridis*[3]. However, there is a description of a greener *D. salina* (KCTC10654BP) from Korea [17] with low cellular *β*-carotene. But 18S rDNA size details are not available for the above Korean strain for comparison. All these factors along with the appearance of *D. viridis/D. peircei* UTEX 2192 close to S118 in *rbc*L phylogeny (clade 1, sub-clade C) and 18S intron phylogeny of *D. peircei* UTEX 2192 by Hajezi et al. [29], emphasizes a need of revisiting the taxonomic identity of all the above reported strains along with the 3 Indian strains using molecular approaches.

GROUP III was formed by 3 strains, S115 (Chennai), S122 (Goa) and S133 (Kutch) allied to *D. viridis*. This further confirms the possibility of the former 2 strains to be Indian isolates of *D. viridis,* while the presence of 2 stigmata in S133 (only at lower salinity) has to be considered for re-examination and for final taxonomic identification.

The remaining 2 strains S125 and S147 were placed in 2 GROUPs (IV and V) as they were more clearly separated from other *Dunaliella* strains on the basis of genetic characters than morpho-physiological traits. Based on the taxonomic key [3] the strain S125 was identified as *D. minuta* (longer pyriform cells) but with clear separate starch granules in pyrenoid differing from *D. minuta*. This strain from Goa salt pan clustered with *D. viridis* (Figures 4 and 6), but with larger divergence values in both (ITS 4.98%, *rbc*L 3.84%) the phylogenies. Further due to the lack of molecular similarity with reported *D. minuta* (NCBI-BLAST analysis of ITS 2, results not given), the identity of the strain was kept in question and placed in GROUP IV. The identity of the Kutch strain S147 was a little confusing but interesting. It resembled *D. tertiolecta* in general morphology and 18S rDNA size (~1820 bp), while grouped with *D. viridis* in ITS phylogeny (Figures 4 &5) and with *D. salina* in *rbc*L phylogeny (Figure 6). It was isolated from a salt pan, having a dominant palmella stage and with a little higher *β*-carotene content (6.7 pg.cell^-1^) in stress, while *D. tertiolecta* was reported as a marine species without a palmella stage in its life cycle [3]. These observations resulted in the grouping of S147 separately as GROUP V and are showing a probability for a new species in the group.

### Diversity in Indian *Dunaliella* strains

Buchheim et al. [9] have reported diverse community formation of *Dunaliella* in heteroclimatic hypersaline soils than in purely aquatic habitats. They hypothesized that external factors like temperature and salinity can enhance diversification and apparently got supporting results from the phylogenetic study of about 30 different isolates of *Dunaliella* (where 3 different morphotypes were characterized), based on 4 genes (18S, 26S, ITS &*rbc*L). Subsequently, Azua-Bustos et al. [40] reported a morphologically distinct, new *Dunaliella* species, *D. atacamensis*, well adapted for sub-aerial life and with higher genetic divergence from its sister species. Our isolates are purely from aquatic habitats, but with high level of environmental fluctuations, especially in salt pans, and showed high divergence when compared to the reported *Dunaliella* species (Clade1 of Figures 4 &6) from NCBI. The geographic distance and isolation of the locations (Figure 1), from where the strains were obtained could be proposed as a reason for the divergence among the above Indian *Dunaliella* isolates. However 100% sequence similarity and morphological resemblance observed between the two isolates S115 and S122 (isolated from Chennai - East coast and Goa - West coast respectively) need to be taken into account.

**Figure 7:**
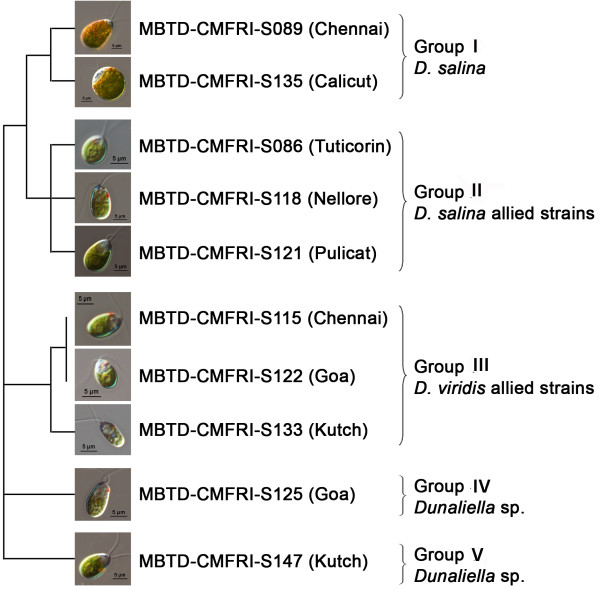
**Schematic representation of diversity of Indian *Dunaliella.*** Grouping was done based on the morphology, 18S rDNA size variation (Figure 3) and ITS and *rbc*L gene phylogenies (Figures 4 &5).

Grouping pattern observed in the reported *Dunaliella* strains from NCBI in the cladistic studies ([27,29], present study) suggests a taxonomic revision of the strains especially when there are comments on confusion regarding the taxonomic status of many reported species. Consequently, Browitzka and Siva [3] have proposed an elaborate morphology/physiology based examination of each strain in conjunction with molecular biology. However, among the 28 morphologically differentiated species [3,8], molecular aspects of only few important ones have been extensively studied and reported, and a very large percentage still remains unexplored genetically. Hence, even after a detailed study based on morphology, physiology and molecular aspects, particularly to avoid misnaming, strain codes were assigned to our isolates which are more appropriate for comparative studies as well as for future communications. Morphological and physiological study precisely groups 6 Indian strains into 2 sections – the carotenogenic Section Dunaliella (S089 and S135) and the non-carotenogenic Section Viridis (S115, S122, S133 and S125) [3,7]. The probability of the remaining 4 strains (S086, S118, S121 and S147) to come under Section Dunaliella is much higher as they are more carotenogenic (S147) and closer to *D. salina* (S086, S118, S121) in molecular analysis. A schematic representation of the diversity in Indian *Dunaliella* is given in Figure 7.

The sequence diversity within the Indian *Dunaliella* strains was distinct when compared to the listed species of *Dunaliella* (ITS region &*rbc*L gene), and shows possibility of the presence of multiple species in the group. Without the knowledge of sexual compatibility between the genotypes, it is not possible to determine whether this diversity is really representing a biological/evolutionary species or merely an intra-specific diversity (9). However, Coleman et al. [41] have demonstrated a concurrence between ITS sequences and mating ability in *Dunaliella* spp. [14]. Since, high level of sequence divergence observed among the Indian *Dunaliella* strains, could be correlated with sexual incompatibility, chances of more species/subspecies with respect to ITS phylogeny seems to be a realistic possibility. Accordingly, the present study proposes the ITS region to be selected as a molecular marker in taxonomic delineation, which is smaller than 18S rRNA gene (with introns) and more diverged than *rbc*L gene.

## Conclusion

The present study clearly shows high diversity within the Indian *Dunaliella* and reliability of 18S rDNA, ITS region and *rbc*L gene sequencing as a molecular tool in species identification and genetic diversity studies. In a recent study, based on morphological parameters Jayapriyan et al. [33] have denoted the presence of 5 species of *Dunaliella* (*D. bioculata*, *D. tertiolecta*, *D. viridis*, *D. minuta* and *D. maritima*) from India (east coast). However in the same study, 18S rDNA species specific fingerprinting using primers of Olmos et al. [31,38] have illustrated the same isolates as completely different species of *Dunaliella* (*D. parva*, *D. bardawil* and an unidentified *Dunaliella* sp.). Hence in the present study, for more clarity on the species lineages, along with morphology and 18S rDNA size, phylogenies based on a more diverse ITS region and a more conserved *rbc*L gene were also included, which otherwise are not available for Indian *Dunaliella*. Consequently, presence of 5 or more species (or sub species), including 2 promising strains of *D. salina* (Section Dunaliella) and 2 *D. viridis*? (Section Viridis) strains, has got confirmed. The genetic characterization further helped in the separation of morphologically similar strains and in the clustering of Indian strains of *Dunaliella* into 5 groups. Similarly, clustering of the reported species in a single clade (Clade 1 with 100% similarity) in a both the phylogenies clearly emphasizes most careful recording of species names [3]. Hence, for resolving the issue prevailing in *Dunaliella* taxonomy and for elucidation of taxonomic species lineation of unknown Indian isolates, it is stressed to have further detailed molecular assessment coupled with additional examination of morphological (based on electron microscopy) and biological traits such as reproductive behavior (asexual- palmella, aplanospores etc.) and sexual compatibility.

## Materials and methods

### Sampling, isolation and culture conditions

Water samples were collected from selected water bodies along the Indian coast during the months of February to July 2009 (Figure 1 & Table 1). Strains were isolated by serial dilution directly or after enrichment for a period of 1 or 2 weeks. Further purification was done with agar plating and picking the single colony to obtain unialgal cultures. *Dunaliella salina* CS265 was purchased from Collection of Living Microalgae, CSIRO, Australia and used as a reference strain. The strain S089 (*D. salina*?, an old isolate from Chennai salt pan) was collected from Phytoplankton (aquaculture live feed) Culture Collection at CMFRI, Kochi. After purification, all the cultures were maintained in 75 ml modified Johnson (J/I) medium [10] in 100 ml flasks with 1.5 M (~200 ppt) NaCl, at temperature 25±1°C and at an irradiance of 40–50 μmol photons m^-2^ s^-1^ supplied by cool white fluorescent lamps on a 12 h light: 12 h dark cycle. All cultures were sub-cultured once in a month basis.

### Microscopy and morphological study

Live culture samples were examined using a Nikon 80i Research microscope (Nikon, Japan) with DIC (Differential Interference Contrast) optics and images were captured using Nikon DSFi 1e camera. Major taxonomic features observed include size, shape and colour of the cell, length of flagella, characteristics of stigma, pyrenoid and chloroplast and other cytoplasmic inclusions like refractile granules. Scalar measurements such as cell length and width, were taken from a minimum of 30 cells from each strain randomly during mid growth phase immediately after fixing the cells with 1% Lugol’s iodine. The descriptive statistics such as minimum, maximum, mean and standard deviation were estimated for the above scalar measurements. One way analysis of variance (ANOVA) was performed using SPSS (Version 10.0) to identify whether there is any statistically significant difference among different *Dunaliella* strains for each character.

### Salinity tolerance study

For salinity tolerance study, different *Dunaliella* strains were cultured in 5 salinity concentrations *viz*., 0.5, 1.5, 2.5, 3.5 and 4.5 M NaCl in 150 ml (250 ml conical flasks) modified Johnson (J/I) medium. Other culture conditions like temperature and light were kept constant as given for normal culture maintenance. Cell characteristics like cell size and colour were examined at late growth phase under DIC microscope (Nikon, Japan). Cell count was taken on every third day using a Neubauer haemocytometer. Cell density was calculated and plotted against days of growth to obtain optimum salinity for each strain.

### *β-*carotene analysis

Beta carotene was estimated under normal (1.5M NaCl, irradiance of 40–50 μmol photons m^-2^ s^-1^) and stressed (3.5M NaCl, irradiance of 100–150 μmol photons m^-2^ s^-1^) growth conditions. Total pigment was extracted from 4 ml culture at late growth phase (25^th^ day) in 4 ml ice cold 100 % acetone. Liquid cultures were centrifuged (8000 rpm, 10 min.), the pellet washed with distilled water and re-suspended in ice cold acetone and left overnight at −20°C until the pellet became colourless. The extract was centrifuged at 5000 rpm for 5 min and absorbance was taken for the supernatant at 454 nm wavelength. Readings were compared with standard curve prepared with synthetic *β-*carotene (Type 1, Sigma, USA) in 100% acetone as described by Hajezi et al. [42]. Cell density was calculated for the same day of extraction and *β-*carotene was calculated per cell in picograms.

### DNA isolation

DNA was isolated from 10 ml liquid culture at late growth phase following modified phenol–chloroform method of Wu et al. [43]. Cells were pelletized by centrifugation at 6000 rpm for 5 minutes, washed in distilled water and re-suspended in 450 μl TEG (25 mM TrisHCl; 10 mM EDTA; 50 mM glucose) buffer (pH 8) with Lysozyme (5 mg/ml) and vortexed with glass beads and then added 50 μl 10% SDS. The tubes were then incubated on ice for 10 min. and added 8 μl Proteinase K (20 mg/ml) and further incubated at 60°C for 60 min in a water bath. Once the cells were lysed completely, the DNA was purified following standard phenol/chloroform extraction and ethanol precipitation [44].

### PCR amplification, sequencing and phylogeny

A gene fragment of 18S rRNA was amplified using conserved primers MA1 & MA2 [5,31]. Reactions were carried out in Veriti Thermal Cycler (Applied Biosystems, US) with a total volume of 25 μl containing PCR buffer at 1× concentration with 1.5 mM MgCl_2_, 0.2 mM each dNTP, 1.5 U of Taq polymerase (Sigma, USA) 5 picomoles of each primer and 25 ng of genomic DNA. Thermal cycling initiated with 3 min at 95°C and then 35 cycles of 30 sec at 95°C, 30 sec at 52°C and 2 min at 72°C. Final extension was for 10 min at 72°C. Amplified products were checked by electrophoresis in 1% agarose gel. The size (bp) of the amplified product was calculated by comparing it with standard molecular weight DNA marker (Step up 100 bp DNA ladder, Merck, India) using the software Image Lab version 3 (Biorad, USA).

Internal transcribed spacer (ITS) region (700 bp), including ITS1, 5.8 S rRNA and ITS2, was amplified using the primers ITS1 and ITS4 (17). Thermal cycling followed an initial denaturation for 3 min at 95°C and 35 cycles of 30 sec at 95°C, 10 sec at 55°C and 45 sec at 72°C followed by a final extension at 72°C for 7 min. Partial (700 bp) region of *rbc*L gene was amplified using the primers *rbc*L 475–497 and *rbc*L 1181–1160 following Nozaki et al. [45] and Assuncao et al. [46]. The reaction mix composition for ITS and rbcL gene were the same as in the case of 18S rRNA gene amplification (given above). Amplified products were tested on 1.5% agarose gel.

All PCR products were purified using *Gen*Elute PCR Cleanup Kit (Sigma, USA) following manufacturer’s instruction. Cycle sequencing was carried out using forward primers (MA1 and *rbc*L 475–497) for 18S rRNA and *rbc*L genes respectively. Whereas the ITS region was sequenced using both forward (ITS1) and reverse (ITS4) primers. Sequences of DNA fragments were imported to BLAST [47] for similarity searches with available database at NCBI GenBank. The sequence was further aligned with the various available sequences (Table 4) of *Dunaliella* spp. and, *Chlamydomonas reinhardtii* (ITS) and *Paulschulzia pseudovolvox* (*rbc*L) as out groups using the CLUSTAL-W algorithm [48] in Bioedit 7.0 (DNA Sequence Analysis Software package). To clearly illustrate grouping pattern in Indian *Dunaliella* isolates, *D. tertiolecta* was out grouped in ITS (Figure 5) phylogeny. Pair wise genetic distances among the different *Dunaliella* species and between the present isolates were calculated based on Kimura 2 parameter model for ITS region and Tamura 3 parameter for *rbc*L gene. The best nucleotide substitution model selection and phylogenetic analysis based on maximum likelihood (with 1000 boot strap replications) were carried out using MEGA 5 [49]. All the sequence information generated in the present study were deposited in the NCBI database (Table 1).

## Competing interests

The authors declare that they have no competing interests.

## Authors’ contributions

KKV and KP conceived the study and wrote the manuscript. KP and CSS did isolation and maintenance of cultures and performed experimental work and data analysis. Molecular data analysis and interpretations were done by LJ. All authors have read and approved the final manuscript.

## References

[B1] Avron M Ed B-AA Dunaliella: Physiology, biochemistry and biotechnology 1992 Boca Raton: CRC press

[B2] Ben-Amtoz A Amos R Industrial production of microalgal cell mass and secondary products –Major industrial species Dunaliella Handbook of Microalgal Culture 2004 USA: Blackwell Publishing 273 280

[B3] Borowitzka MA Siva CJ The taxonomy of the genus Dunaliella (Chlorophyta, Dunaliellales) with emphasis on the marine and halophilic species J Appl Phycol 2007 19 567 590 10.1007/s10811-007-9171-x

[B4] Tafreshi HA Shariati M Dunaliella biotechnology: methods and applications J Appl Microbio 2009 107 14 35 10.1111/j.1365-2672.2009.04153.x 19245408

[B5] Olmos SJ Ochoa L Paniagua-Michel J Contreras R DNA fingerprinting differentiation between β- carotene hyperproducer strains of Dunaliella from around the world Saline Systems 2009 5 5 10.1186/1746-1448-5-5 19563682 PMC2710335

[B6] Oren A A hundred years of Dunaliella research: 1905–2005 Saline systems 2005 1 1 14 10.1186/1746-1448-1-1 16176593 PMC1224875

[B7] Preisig HR Avron M, Ben-Amtoz A Morphology and taxonomy Dunaliella: Physiology, Biochemistry and biotechnology 1992 Boca Raton: CRC Press 1 15

[B8] Gonzalez MA Gomez PI Polle JEW Ben-Amtoz A, Polle JEW, Subba Rao DV Taxonomy and phylogeny of the genus Dunaliella The alga Dunaliella, biodiversity, physiology, genomics and biotechnology 2009 Enfield: Science Publishers 15 44

[B9] Buchheim MA Kirkwood AE Buchheim JA Verghese B Henly WJ Hypersaline soil supports a diverse community of Dunaliella (Chlorophyceae) J Phycol 2010 46 1038 1047 10.1111/j.1529-8817.2010.00886.x

[B10] Borowitzka MA Borowitzka LJ Borowitzka MA, Borowitzka LJ Dunaliella Microalgal Biotechnology 1988 New York: Cambridge University press 27 58

[B11] Spaulore P Joanni S Cassan C Duran E Isambert A Commercial applications of microalgae J Biosci Bioeng 2005 102 87 96 10.1263/jbb.101.87 16569602

[B12] Kleinegris DMM Janssen M Brandenberg WA Wijfells RH The selectivity of milking Dunaliella salina Mar Biotechnol 2010 12 14 23 10.1007/s10126-009-9195-0 19475448 PMC2816252

[B13] Gomez PI Gonzaléz MA Genetic polymorphism in eight Chilean strains of the carotenogenic microalga Dunaliella salina Teodoresco (Chlorophyta) Biol Res 2001 34 23 30 11471520 10.4067/s0716-97602001000100012

[B14] Gomez PI Gonzalez MA Genetic variation among seven strains of Dunaliella salina (Chlorophyta) with industrial potential, based on RAPD banding patterns and on nuclear ITS rDNA sequences Aquaculture 2004 233 149 162 10.1016/j.aquaculture.2003.11.005

[B15] Raja R Hema IS Balasubramanyam D Rengasamy R Exploitation of Dunaliella for beta-carotene production Appl Microbiol Biotechnol 2007 74 517 523 10.1007/s00253-006-0777-8 17225103

[B16] Raja R Hema IS Balasubramanyam D Rengasamy R PCR-identification of Dunaliella salina (Volvocales, Chlorophyta) and its growth characteristics Microbiol Res 2007 162 168 176 10.1016/j.micres.2006.03.006 16697630

[B17] Polle JEW Struwe L Jin E Identification and characterization of a new strain of the unicellular green alga Dunaliella salina (Teod.) from Korea J Microbiol Biotchnol 2008 18 821 827 18633277

[B18] Mishra A Jha B Isolation and characterization of extracellular polymeric substances from microalga Dunaliella salina under salt stress Biores Technol 2009 100 3382 3386 10.1016/j.biortech.2009.02.006 19272770

[B19] Mishra A Jha B Cloning differentially expressed salt induced cDNAs from Dunaliella salina super saturated salt stress using subtractive hybridization Bot Mar 2011 54 189 193

[B20] Eyden BP Light and electron microscopic study of Dunaliella primolecta Butcher (Volvocida) J Protozool 1975 22 336 344

[B21] Hoshaw RW Malouf LY Ultrastructure of the green flagellate Dunaliella tertiolecta (Chlorophyceae, Volvocales) with comparative notes on three other species Phycologia 1981 20 199 206 10.2216/i0031-8884-20-2-199.1

[B22] Uriarte I Farias A Hawkins AJS Bayne BL Cell characteristics and biochemical composition of Dunaliella primolecta Butcher conditioned at different concentrations of dissolved nitrogen J Appl Phycol 1993 5 447 453 10.1007/BF02182737

[B23] Gonzalez MA Coleman AW Gomez PI Montoya R Phylogenetic relationship among various strains of Dunaliella (chlorophyceae) based on nuclear ITS rDNA sequences J Phycol 2001 37 604 611 10.1046/j.1529-8817.2001.037004604.x

[B24] Teodoresco EC Organisation et developpement du Dunaliella, nouveau genre de Volvocacee- Polyblepharidee Beih z Bot Centralbl 1905 Bd XVIII 215 232

[B25] Massyuk NP Morphology, Taxonomy, Ecology and Geographic distribution of the Genus Dunaliela Teod. and prospectus for its potential utilization 1973 Kiev: Naukova Dumka (In Russian)

[B26] Oliveira L Bisalputra T Anita NJ Ultra structural observation of the surface coat of Dunaliella tertiolecta from staining with cataionic dyes and enzyme treatments New Phytol 1980 85 385 392 10.1111/j.1469-8137.1980.tb03177.x

[B27] Gonzalez MA Gomez PI Montoya R Comparison of PCR-RFLP nalysis in the ITS region with morphological criteria of various strains of Dunaliella J Appl Phycol 1999 10 573 580

[B28] Chen H Jiang JG Osmotic responses of Dunaliella to the changes of salinity J Cell Physiol 2009 219 251 258 10.1002/jcp.21715 19202552

[B29] Hajezi MA Barzegari A Gharajeh NH Hajezi MS Introduction of a novel 18S rDNAgene arrangement along with distinct ITS region in the saline water microalga Dunaliella Saline Systems 2010 6 4 10.1186/1746-1448-6-4 20377865 PMC2867797

[B30] Bornet B Antoine E Bardouil M Baut MC ISSR as new markers for genetic characterization and evaluation of relationships among phytoplankton J Appl Phycol 2004 16 285 290

[B31] Olmos SJ Paniagua MJ Contreras FR Molecular identification of Dunaliella sp. utilizing the 18S rDNA gene Lett Appl Microbiol 2000 30 80 84 10.1046/j.1472-765x.2000.00672.x 10728567

[B32] Ramos AA Polle J Trau D Cushman JC Jin ES Varela CJ The unicellular green alga Dunaliella salina as a model for abiotic stress tolerance: genetic advances and future perspectives Algae 2011 26 3 20 10.4490/algae.2011.26.1.003

[B33] Jayapriyan KR Rajkumar R Sheeja L Nagaraj S Divya S Rengasamy R Discrimination between the morphological and molecular identification in the genus Dunaliella Int J Cur Res 2010 8 73 78

[B34] Massyuk NP New taxa of the genus Dunaliella Teod I Ukr Bot Zh 1973 30 175

[B35] Ginzburg M Ginzburg BZ Ion and glycerol concentrations in 12 siolates of Dunaliella J Exp Bot 1985 36 1064 1074 10.1093/jxb/36.7.1064

[B36] Coesel SN Baumgartner AC Teles LM Ramos AA Henriques NM Cancela L Varela JCS Nutrient limitation is the main regulatory factor for carotenoid accumulation and for Psy and Pds steady state transcript levels in Dunaliella salina (Chlorophyta) exposed to high light and salt stress Marine Biotech 2008 10 602 611 10.1007/s10126-008-9100-2 18449600

[B37] Wilcox LW Lewis A Fuerst PA Floyd GL Group I introns within the nuclear-encoded small-subunit rRNA gene of three green algae Mol Biol Evol 1992 9 1103 1118 1435237 10.1093/oxfordjournals.molbev.a040781

[B38] Olmos SJ Paniagua MJ Contreras FR Trujillo L Molecular identification of β-carotene hyperproducing strains of Dunaliella from saline environments using species specific oligonucleotides Biotechnol Letters 2002 24 365 369 10.1023/A:1014516920887

[B39] Coleman AW Mai JC Ribosomal DNA ITS-1 and ITS-2 sequence comparisons as a tool for predicting genetic relatedness J Mol Evol 1997 45 168 177 10.1007/PL00006217 9236277

[B40] Azua-Bustos A Gonzalez-Silva C Salas L Palma RE Vicuna R A novel sub-aerial Dunaliella species growing on cave spider webs on Atacama Desert Extremophiles 2010 14 443 452 10.1007/s00792-010-0322-7 20623153

[B41] Coleman AW The significance of coincidence between evolutionary landmarks found in mating affinity and DNA sequence Protist 2000 151 1 9 10.1078/1434-4610-00002 10896128

[B42] Hajezi MA Lamarliere C Rocha JMS Vermue M Tramper J Wijffels RH Selective extraction of carotenoid from the alga Dunaliella salina with retention of viability Biotechnol Bioeng 2002 79 29 36 10.1002/bit.10270 17590929

[B43] Wu X Zarka A Boussiba S A simplified protocol for preparing DNA from filamentous Cyanobacteria Plant Mol Biol 2000 18 385 392 10.1007/BF02825067

[B44] Sambrook J Fritsch EF Maniatis T Molecular Cloning: a Laboratory Manual 1989 Cold Spring Harbor Laboratory Press: Cold Spring Harbor

[B45] Nozaki H Ito M Sano R Uchida H Watanabe MM Takahashi H Phylogenetic relationships within the colonial Volvocales (Chlorophyta) inferred from rbcL gene sequence data J Phycol 1995 31 970 979 10.1111/j.0022-3646.1995.00970.x

[B46] Assuncao P Jaen-Molina R Caujape-Castells J Jara A Carmona L Freijanes K Mendoza H Phylogenetic position of Dunaliella acidophila (Chlorophyceae) based on ITS and rbcL sequences Dig J Appl Phycol 2011 10.1007/s10811-001-9676-1

[B47] Altschul SF Gish W Miller W Myers EW Lipman DJ Basic local alignment search tool J Mol Biol 1990 215 403 410 2231712 10.1016/S0022-2836(05)80360-2

[B48] Thompson JD Higgins DG Gibson TJ CLUSTAL W: Improving the sensitivity of progressive multiple sequence alignment through sequence weighing, positions specific gap penalties and weight matrix choice Nucleic Acids Res 1995 22 4673 4680 7984417 10.1093/nar/22.22.4673 PMC308517

[B49] Tamura K Peterson D Peterson N Stecher G Nei M Kumar S MEGA5: Molecular evolutionary genetics analysis using maximum likelihood, evolutionary distance, and maximum parsimony methods Mol Biol Evolution 2011 28 2731 2739 10.1093/molbev/msr121 PMC3203626 21546353

